# Effectiveness and Utility of Genetic Testing in Establishing a Diagnosis of Hereditary Transthyretin Amyloidosis †

**DOI:** 10.3390/jcm14196821

**Published:** 2025-09-26

**Authors:** Akash Singh, John Wyatt, Marie Théaudin, Chafic Karam, David Kasper, Berthold Streubel, Karen Frascello, Antoine Bondue

**Affiliations:** 1 Alnylam Pharmaceuticals, London SL6 1DA, UK; 2 Alnylam Pharmaceuticals, Cambridge, MA 02142, USA; jowyatt@alnylam.com (J.W.); kfrascello@alnylam.com (K.F.); 3 Service of Neurology, Neurosciences Department, Lausanne University Hospital (CHUV), University of Lausanne, 1011 Lausanne, Switzerland; marie.theaudin@chuv.ch; 4 Department of Neurology, University of Pennsylvania, Philadelphia, PA 19104, USA; chafic.karam@pennmedicine.upenn.edu; 5 ARCHIMED Life Science GmbH (ARCHIMEDlife), 1110 Vienna, Austria; d.kasper@archimedlife.com; 6 Clinical Institute of Pathology, Medical University of Vienna, 1090 Vienna, Austria; berthold.streubel@meduniwien.ac.at; 7 Department of Cardiology, Hôpital Universitaire de Bruxelles, Hôpital Erasme, Université Libre de Bruxelles, 1070 Brussels, Belgium; antoine.bondue@hubruxelles.be

**Keywords:** ATTRv, transthyretin, amyloidosis, TTR, genetic testing

## Abstract

**Background/Objectives:** Hereditary transthyretin amyloidosis (ATTRv) is a progressive and fatal disease with >130 known underlying variants in the *TTR* gene. We describe the utility of two no-charge genetic testing programs in identifying *TTR* variants in participants across Europe/Middle East and North America, respectively. **Methods:** Eligible adult participants in GeneAct^®^ and Alnylam Act^®^ had a family history or clinical suspicion of ATTRv. Testing was performed using gene panels for neuropathies or cardiomyopathy, or a single-gene *TTR* test. Diagnostic yield was defined as one pathogenic/likely pathogenic variant in *TTR*. **Results:** Overall, 2713 and 89,760 participants were tested in GeneAct^®^ and Alnylam Act^®^. Genetic diagnosis was established in 95 and 4297 participants, respectively, resulting in a diagnostic yield of 3.5% and 4.8%. V122I (p.V142I) was the most common variant, identified in 34 of these participants in GeneAct^®^ and 3299 in Alnylam Act^®^. Cardiac and neurologic signs/symptoms were the most common manifestations across both programs, as reflected in the specialties ordering tests in Alnylam Act^®^ (cardiology, 29.1%; neurology, 31.5%). **Conclusions:** These data highlight the importance of genetic testing for early identification of ATTRv, especially among patients with cardiac and neuropathy symptoms. Genetic testing has the potential to improve diagnostic timeframes and outcomes in ATTRv.

## 1. Introduction

Transthyretin amyloidosis (ATTR) is an underdiagnosed, progressive, and fatal disease caused by the accumulation of toxic transthyretin (TTR) amyloid fibrils in multiple tissues and organs, including the peripheral nerves and heart [1,2,3]. Two forms of ATTR exist: wild-type ATTR (ATTRwt), which occurs with aging and is caused by the amyloidogenic deposition of misfolded wild-type TTR, and hereditary ATTR (ATTRv), which is caused by variants in the *TTR* gene that predispose TTR to misfold and accumulate as amyloid fibrils [1,2,4].

ATTRv has an autosomal dominant pattern of inheritance with incomplete penetrance [5]. More than 130 unique variants have been identified in the *TTR* gene to date, which differ in geographic distribution and penetrance, and manifest with various clinical presentations including neuropathy and cardiomyopathy [2,6,7]. The non-specific and variable constellation of symptoms, coupled with the relative rarity of ATTRv, can lead to misdiagnosis and/or delays in clinical diagnosis of several years [8,9], and diagnosis ultimately requires a high level of clinical suspicion [10]. However, because ATTRv can progress rapidly after onset of symptoms, prompt diagnosis is critical for early treatment intervention that may increase survival and/or prevent irreversible deterioration of physical function and quality of life [11,12,13]. ATTRv identification is also the first step for further cascade genetic testing and counseling of the family members of affected individuals, offering a valuable tool for risk stratification, follow-up, and early diagnosis [14].

Genetic testing is recommended in patients with a diagnosis of ATTR to distinguish ATTRv and ATTRwt [10,14,15]. In addition, testing can lead to genetic diagnosis in patients with clinical suspicion of ATTRv, for example, in those with polyneuropathy of unknown etiology, and asymptomatic individuals with a family history of disease. Importantly, it is necessary to identify variants in the *TTR* gene that may inform management and treatment strategies, with clinical assessments in carriers recommended to begin 5–10 years before predicted age of onset [10,14,15,16,17,18]. However, genetic testing is not widely established or implemented and can be limited by poor access to testing services, reimbursement issues, limited expertise, and long test turnaround times [14,15,19], meaning there is a need to optimize genetic testing as part of improvements in the care pathway of ATTR.

In this study, we set out to describe the effectiveness and utility of two no-charge genetic testing programs, GeneAct^®^ and Alnylam Act^®^, in identifying *TTR* variants in participants with suspected ATTRv or a family history of ATTRv across Europe, the Middle East, and North America.

## 2. Materials and Methods

### 2.1. Study Design

GeneAct^®^ is a genetic testing service that was available in several countries across Europe (Austria, Belgium, Bulgaria, Cyprus, Czechia, France, Germany, Greece, Ireland, Luxembourg, Malta, Poland, Romania, Slovenia, Switzerland, and the United Kingdom) and the Middle East (Bahrain, Kuwait, Oman, Qatar, Saudi Arabia, and the United Arab Emirates), and it is supported and funded by Alnylam Pharmaceuticals (note that in Germany, the testing service was supported, but not funded, by Alnylam Pharmaceuticals), which offers physicians access to genetic tests to identify individuals with *TTR* variants at no cost. Blood samples for genetic analysis were collected on CE-certified dried blood spot cards. Genomic DNA was isolated from dried blood spot cards using the Chemagic™ 360 Instrument and Chemagic DNA Blood Spot 3 kit (both PerkinElmer, Shelton, CT, USA). The transcript NM_000371.4 served as reference sequence for the *TTR* gene. Testing in the GeneAct^®^ program was conducted using a Sanger sequencing-based single-gene test (ARCHIMEDlife, Vienna, Austria) and included analysis of the entire *TTR* gene (exons 1–4) and flanking intronic regions (+/– 20 base pairs) for single nucleotide variants and small deletions/insertions. Potentially pathogenic variants were compared with known variants in the PubMed, HGMD^®^, ClinVar, and MASTERMIND databases. Unpublished variants were interpreted according to the American College of Medical Genetics/Association for Molecular Pathology 2015 guidelines for interpretation of sequence variants [20].

Alnylam Act^®^ is a no-charge, third-party, genetic testing and counseling program for individuals in the USA and Canada with a family history or suspected diagnosis of ATTRv [21], sponsored by Alnylam Pharmaceuticals. Testing in Alnylam Act^®^ was conducted according to physician’s choice of the following tests used alone or in combination: a neuropathies panel of 82 genes including TTR; a cardiomyopathies panel of 102 genes including TTR; and a single-gene test for TTR variants. Samples for testing included whole blood, saliva, and buccal swab. In both programs, physicians had the option of providing information on clinical signs/symptoms on the test requisition form.

### 2.2. Study Population

Individuals eligible for testing in the GeneAct^®^ program were ≥18 years of age with either a family history of ATTRv or suspicion of ATTRv based on ≥1 of the following findings: sensory neuropathy; motor neuropathy; autonomic dysfunction; heart disease; bilateral carpal tunnel syndrome; lumbar spinal stenosis; renal abnormalities; ocular changes; chronic inflammatory demyelinating polyneuropathy-atypical; motor neuron disease-atypical; gait disorders; unexplained weight loss; and imaging/biopsy positive.

Individuals eligible for testing in the Alnylam Act^®^ program were ≥18 years of age with ≥1 ATTRv high index of suspicion indicator that included family history of ATTRv amyloidosis; positive imaging consistent with amyloid (technetium, cardiac magnetic resonance, strain echo); or positive biopsy for TTR amyloid; or ≥2 ATTRv index of suspicion indicators that included sensory and/or motor neuropathy (e.g., neuropathic pain, altered sensation, numbness and tingling, muscle weakness, impaired balance, difficulty walking, carpal tunnel syndrome-associated neuropathy, electromyography/nerve conduction study abnormalities); autonomic dysfunction (e.g., nausea and vomiting, changes in gastrointestinal motility, orthostatic hypotension, sexual dysfunction, bladder dysfunction); heart disease (e.g., cardiomyopathy, restrictive physiology, hypertrophy, arrhythmias, conduction abnormalities, heart failure, abnormal cardiac imaging); musculoskeletal indicators (history of carpal tunnel syndrome, back pain/lumbar spinal stenosis, rotator cuff injury); renal abnormalities (e.g., renal insufficiency and/or proteinuria); and ocular changes (e.g., vitreous opacity, glaucoma, dry eyes, ocular amyloid angiopathy, retinal detachment).

### 2.3. Data Analysis

Descriptive analyses were performed on data collected between January 2022 and May 2024 for GeneAct^®^, and between August 2017 and May 2023 for Alnylam Act^®^. The available data were limited to the results of the genetic test, the information on the test requisition form for Alnylam Act^®^ and the patient consent form for GeneAct^®^, and any other details the physician provided. Diagnostic yield was defined as one pathogenic or likely pathogenic variant in the *TTR* gene. Results by *TTR* variant were analyzed for Alnylam Act^®^ but not for GeneAct^®^ because of the small sample size in the latter study. Figure S1 showing the distribution of variants within the *TTR* gene was generated using Mutation Mapper software (Portal Version: v6.3.6; db Version: 2.14.2).

## 3. Results

### 3.1. TTR Positivity

**Figure 1:**
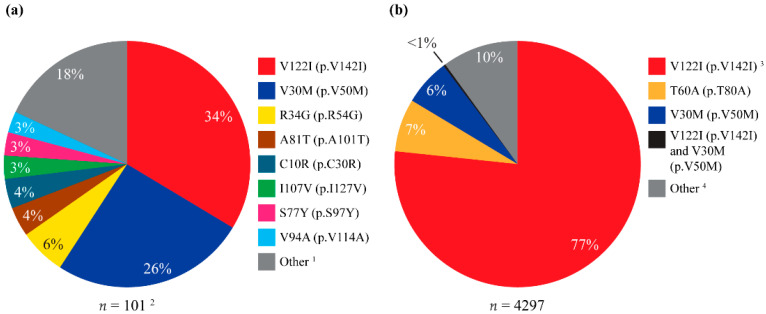
Variants identified in the *TTR* gene in (**a**) GeneAct^®^ and (**b**) Alnylam Act^®^. ^1^ Other variants were D74H (p.D94H), T60A (p.T80A), V71A (p.V91A) (all *n* = 2), A25S (p.A45S), R103H (p.R123H), p.R5H, E89Q (p.E109Q), E89K (p.E109K), E7* (p.E27*), E54Q (p.E74Q), E61G (p.E81G), G67V (p.G87V), H31N (p.H51N), S100T (p.S120T), and S77F (p.S97F) (all *n* = 1). ^2^ Includes 5 variants of uncertain significance that were identified in 6 symptomatic participants but were excluded from the calculation of diagnostic yield. ^3^ Includes 4 participants with 2 variants: V122I (p.V142I) and R103H (p.R123H), V122I (p.V142I) and I107V (p.I127V), V122I (p.V142I) and I68L (p.I88L), and V122I (p.V142I) and T60I (p.T80I) (all *n* = 1). ^4^ Includes L58H (p.L78H) (*n* = 63), P64L (p.P84L) (*n* = 61), I107V (p.I127V) (*n* = 36), I68L (p.I88L) (*n* = 29), S77T (p.S97T) (*n* = 25), and P24S (p.P44S) (*n* = 21) (see Table S2 for the full list of other variants identified). * indicates a stop codon. Percentages may not add up to 100 due to rounding.

Samples from 2713 participants across 22 countries were analyzed in the GeneAct^®^ program between January 2022 and May 2024. A total of 101 participants had a positive test for a *TTR* variant; this included 6 participants with variants of uncertain significance who were excluded from the diagnostic yield calculation. A total of 95 participants had a positive test for a pathogenic or likely pathogenic variant in the *TTR* gene (all in the heterozygous state), resulting in a 3.5% diagnostic yield; 18 unique pathogenic or likely pathogenic variants were identified (Table S1). The most common variants identified were V122I (p.V142I) and V30M (p.V50M), which were found in 34 (33.7%) and 26 (25.7%) participants with a positive test, respectively (Figure 1a). Identified variants occurred throughout the *TTR* gene (Figure S1), with the largest number of unique variants detected in exon 3. Six symptomatic participants had 5 *TTR* variants of uncertain significance (Table S1).

Overall, 89,760 participants from the USA/Canada were tested in the Alnylam Act^®^ program between August 2017 and May 2023, with 11,149 (12.4%) participants having a positive test for any gene across the panels used for analysis (Table S3). A total of 4297 participants had a positive test for a pathogenic or likely pathogenic variant in the *TTR* gene, resulting in a 4.8% diagnostic yield. Of these participants, 162 (3.8%) had pathogenic or likely pathogenic *TTR* variants in the homozygous state. Among participants with a confirmed family history, diagnostic yield was 32.2%. V122I (p.V142I) was the most commonly identified variant (*n* = 3299 [76.8%]; Figure 1b; Table S2), followed by T60A (p.T80A) (*n* = 292, 6.8%) and V30M (p.V50M) (*n* = 271, 6.3%); 434 (10.1%) participants with a positive test had other TTR variants, while one participant tested positive for both V122I (p.V142I) and V30M (p.V50M) (0.02%). An additional 166 participants had *TTR* variants of uncertain significance (Table S2).

### 3.2. Baseline Characteristics

**Figure 2:**
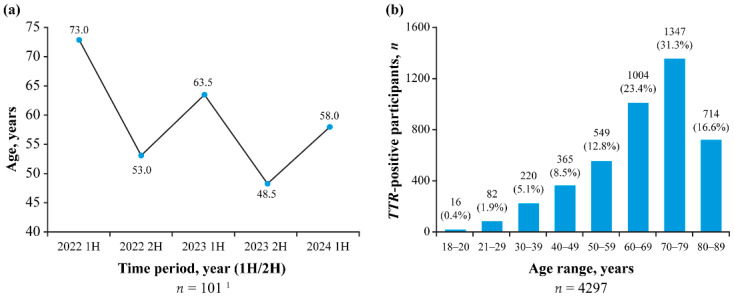
Median age of participants with *TTR* variants at genetic testing in (**a**) GeneAct^®^ and (**b**) *TTR* positivity by age range in Alnylam Act^®^. ^1^ Includes 6 participants with variants of uncertain significance. 1H = first half of the year; 2H = second half of the year.

Mean (standard deviation [SD]) age at testing in GeneAct^®^ was 65.0 (15.9) years for all participants (63.8% male) and 57.2 (18.2) years for participants who tested *TTR*-positive (56.4% male); median age at testing decreased over the duration of the analysis period, dropping from 73.0 years in the first half of 2022 to 58.0 years in the first half of 2024 (Figure 2a). In GeneAct^®^, of the 101 participants with a positive test for a *TTR* variant (including the six symptomatic participants with variants of uncertain significance), 61 participants (60.4%) had a family history of ATTRv. Three of these participants were confirmed by their physician as being asymptomatic.

The mean (SD) age of participants in Alnylam Act^®^ at testing was 60.5 (16.5) years for the overall population and 65.6 (14.8) years for those who tested *TTR*-positive, with the highest rates of *TTR* positivity observed among those aged 60–69 years (23.4%) and 70–79 years (31.3%) (Figure 2b).

Despite the finding that Black or African American participants represented only 14.3% of the overall population tested in Alnylam Act^®^, 61.4% of those testing positive for variants in the *TTR* gene were Black or African American. Almost all (98.1%; *n* = 2590) Black or African American TTR-positive participants had the V122I (p.V142I) variant. Of all *TTR*-positive participants, 3533 (82.2%) were individuals with a suspected ATTRv diagnosis versus 764 (17.8%) with only a family history of ATTRv. Of participants with only a family history of ATTRv, 451 (59.0%) had the V122I (p.V142I) variant, while 108 (14.1%), 85 (11.1%), and 120 (15.7%) had p.T80A, V30M (p.V50M), and “other” *TTR* variants, respectively. Data on whether these participants with family history were symptomatic/asymptomatic were not available.

Demographics and baseline characteristics for participants (all and *TTR*-positive) in GeneAct^®^ and Alnylam Act^®^ are shown in Table 1.

### 3.3. Presenting Signs and Symptoms

**Figure 3:**
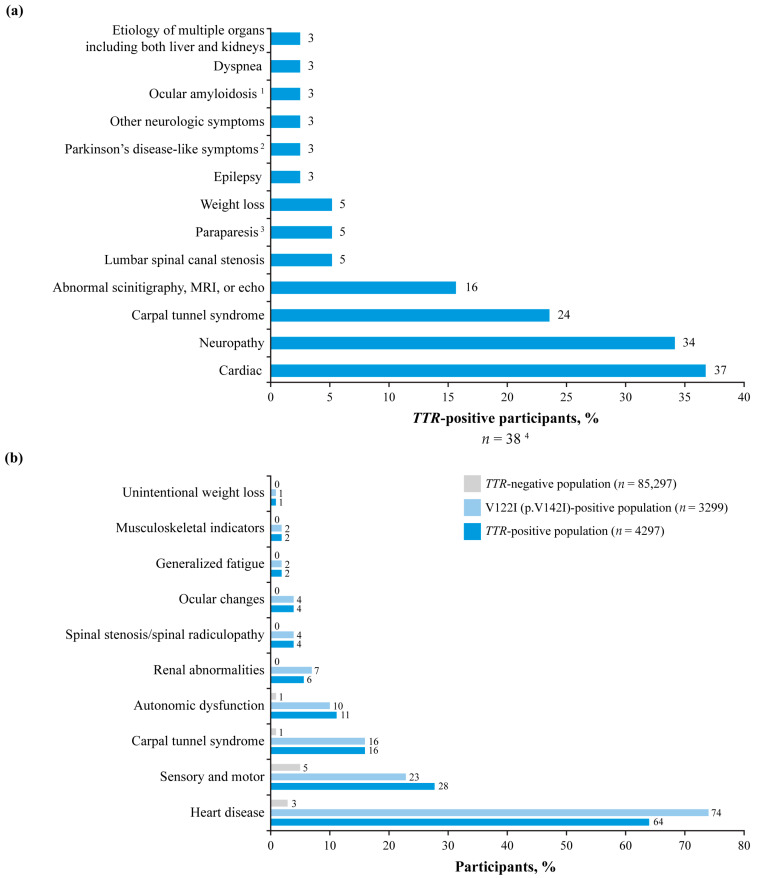
Presenting signs or symptoms in participants with *TTR* variants in (**a**) GeneAct^®^; and in (**b**) *TTR*-positive, *TTR* V122I (p.V142I) variant-positive, and *TTR*-negative participants in Alnylam Act^®^. ^1^ Confirmed cases of ocular amyloidosis where a pathogenic or likely pathogenic *TTR* variant is present. ^2^ A101T was associated with Parkinson’s disease-like symptoms. ^3^ D74H (p.D94H) was associated with paraparesis. ^4^ Data available for 38 of 101 participants with variants in the *TTR* gene. Multiple symptoms may be reported by the same patient. MRI = magnetic resonance imaging.

The most commonly reported clinical manifestations among GeneAct^®^ participants testing positive for a *TTR* variant and with clinical data available (*n* = 38) were cardiac (36.8%) and neuropathy (34.2%) signs or symptoms, followed by carpal tunnel syndrome (23.7%) (Figure 3a). Other reported manifestations included renal, hepatic, musculoskeletal, and ocular abnormalities.

The most common presentation among *TTR*-positive participants in Alnylam Act^®^ was heart disease, which occurred in 64.0% of all *TTR*-positive participants and 74.1% of those with the V122I (p.V142I) variant (Figure 3b; Table S4). Other commonly reported signs and symptoms in *TTR*-positive participants were neurologic (27.8%) and autonomic (11.2%) manifestations (Figure 3b). Data for diagnostic yields according to individual signs and symptoms are shown in Table S5. Family history of ATTRv, positive biopsy for amyloid, and positive imaging had the highest diagnostic yields overall and in participants with a positive *TTR* result. In the latter group, unintentional weight loss, heart disease, carpal tunnel syndrome, renal abnormalities, and generalized fatigue had yields >5%, while musculoskeletal disorders had a yield <2%.

### 3.4. Test Provider Specialties and Types of Testing

**Figure 4:**
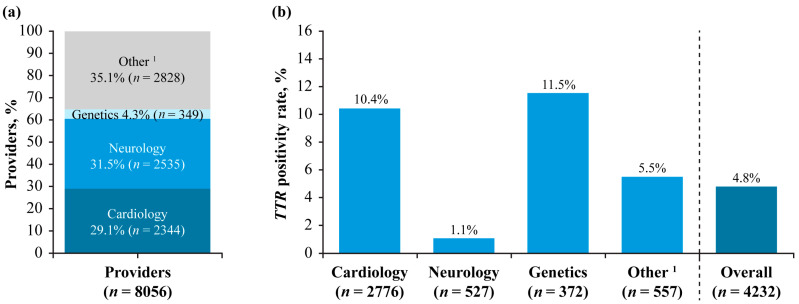
(**a**) Number of test providers by specialty and (**b**) *TTR* positivity rate by specialty in Alnylam Act^®^. ^1^ Other specialties included oncology, primary care, obstetrics, gynecology, pathology, surgery, and emergency medicine.

**Figure 5:**
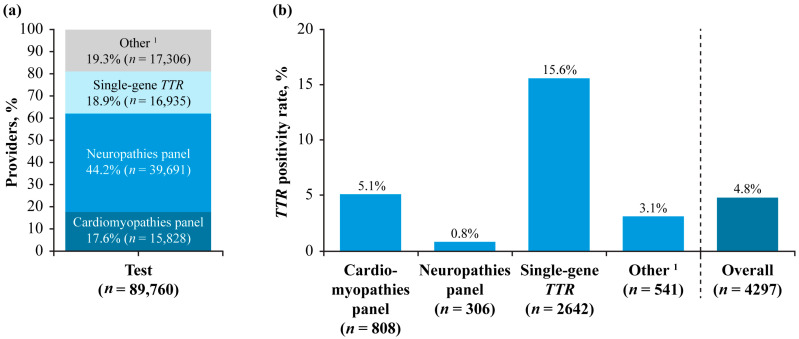
(**a**) Number of tests performed by test type and (**b**) *TTR* positivity by test type in Alnylam Act^®^. ^1^ Combinations of the three test types.

Specialties using the Alnylam Act^®^ program were cardiology (29.1%), neurology (31.5%), genetics (4.3%), and others (35.1%; Figure 4a). Although test providers specializing in neurology were the most common type of provider, the participants referred by neurology specialists had the lowest *TTR* positivity rate (1.1%; Figure 4b). In contrast, participants referred for testing by providers specializing in genetics or cardiology had the highest *TTR* positivity rates (11.5% and 10.4%, respectively; Figure 4b). The neuropathies panel was the most performed test in Alnylam Act^®^ (44.2%; Figure 5a), with an overall positivity rate of 10.2%, but it had the lowest *TTR* positivity rate (0.8%; Figure 5b), while the single-gene *TTR* test had the highest positivity rate of the individual tests (15.6%).

## 4. Discussion

In this study, the utility of the GeneAct^®^ and Alnylam Act^®^ programs for the genetic diagnosis of ATTRv was assessed among >90,000 participants with a family history or suspected ATTRv in 24 countries across Europe, the Middle East, and North America. Diagnostic yields (i.e., the proportion of participants with a pathogenic or likely pathogenic *TTR* variant) of 3.5% and 4.8% (GeneAct^®^ and Alnylam Act^®^, respectively) were consistent with the *TTR* positivity rates reported in other studies [22,23,24,25,26,27,28]. Among participants from North America, older individuals generally had higher rates of *TTR* positivity, while data from participants in Europe/Middle East revealed a trend toward a decreasing median age at genetic diagnosis between 2022 and 2024, possibly reflecting higher disease awareness and/or awareness of the testing program in these regions.

In both programs, participants who tested positive for *TTR* variants had a diverse range of manifestations, including cardiac signs and symptoms, sensory and autonomic neuropathy, other neurologic and musculoskeletal symptoms, and symptoms due to abnormalities with other organs, all of which are consistent with the phenotypic heterogeneity of ATTRv [1,7]. Neurologic signs and symptoms included paraparesis and Parkinson’s disease-like symptoms, both of which are rare manifestations of ATTRv with polyneuropathy (ATTRv-PN). These symptoms were associated with the pathogenic/likely pathogenic TTR variants D74H (p.D94H) and A81T (p.A101T), respectively.

The V122I (p.V142I) variant was the most common variant reported across both programs, and this is consistent with findings from other studies in Europe and the USA [2,29,30]. Differences were observed between Europe/Middle East and North America, however, with V122I (p.V142I) (34%) and V30M (p.V50M) (26%) being the most common variants in Europe/Middle East, compared with V122I (p.V142I) (77%) being the predominant variant in North America. In addition, heart disease was the most common presentation among *TTR*-positive participants in both programs, although this was driven by the high incidence of heart disease among participants with the V122I (p.V142I) variant, and this finding is consistent with previously reported associations between V122I (p.V142I) and heart failure, atrial fibrillation, and hospitalization [31,32,33]. The incidence of heart disease at presentation was considerably lower in participants with other variants, including T60A (p.T80A) and V30M (p.V50M). This is consistent with previous studies, in which both the T60A (p.T80A) and V30M (p.V50M) variants are predominantly associated with neuropathy at presentation [7,34,35]; this was seen in Alnylam Act^®^ for participants with V30M (p.V50M). However, an earlier study in the UK reported a dominant cardiac phenotype at diagnosis associated with T60A (p.T80A) [36]. In Alnylam Act^®^, a family history of ATTRv was reported less commonly with V122I than with other variants. Cardiac signs/symptoms that were reported among participants with the V122I (p.V142I) variant in GeneAct^®^ included cardiomyopathy, heart failure, heart disease, cardiac amyloidosis, and ATTR with cardiomyopathy (ATTR-CM).

In North America, *TTR* positivity rate was highest in participants referred by providers specializing in genetics or cardiology, which may be related to a higher use of pre-test selection modalities among these providers, including the use of technetium cardiac imaging and cardiac biopsy. By contrast, low *TTR* positivity rates among participants referred by neurologists may reflect recommendations to conduct *TTR* genetic testing for all patients with symptoms of rapidly progressing neuropathy, even in the absence of red-flag signs of ATTR [16]. The low *TTR* positivity rates may also reflect findings of previous studies reporting that neurologists may use such gene panels to investigate idiopathic neuropathy and to exclude other potential diagnoses [16], as supported by the overall positivity rate of 10% observed with use of the neuropathy panel in Alnylam Act^®^.

The Alnylam Act^®^ program included use of both gene panels and single-gene tests, with single-gene tests resulting in the highest *TTR* positivity rates. Single-gene testing, e.g., with Sanger sequencing-based methods, may be particularly useful in those individuals for whom there is clinical suspicion of ATTR, because it may be more cost-effective and easier to implement than use of gene panels or next-generation sequencing.

In addition to facilitating earlier diagnosis in patients with suspected ATTRv [19,37,38], genetic testing may be valuable in patients with similar clinical features to ATTRv but who have not responded to therapy for that condition [39]. Achieving an early diagnosis of ATTRv may enable initiation of treatment with disease-modifying therapy for which multiple options are now available, including the RNA interference therapeutics patisiran and vutrisiran, the TTR stabilizers tafamidis and acoramidis, and the antisense oligonucleotides eplontersen and inotersen [1,12,40,41,42,43,44].

There are limitations associated with our study. Firstly, it was not mandatory for the referring physician to provide information on symptoms on the referral form for genetic testing. Therefore, details on symptoms were not available for all participants and it could not be concluded that participants were asymptomatic unless stated. Limited availability of data on clinical characteristics and their association with pathogenic/likely pathogenic variants somewhat limits the ability to make recommendations on which patients should be tested, although cardiac and some neurologic manifestations were frequent characteristics among those testing positive. For example, however, additional clinical information on participants in our study with signs and symptoms of carpal tunnel syndrome may have helped to characterize the large number of individuals with carpal tunnel syndrome with possible ATTRv in practice who should undergo testing. It is also unknown what proportion of patients with a positive genetic test subsequently had a clinical diagnosis of ATTRv. In addition, there is a lack of information on the penetrance of identified *TTR* variants within families, the age of other family members, and whether those family members were subsequently tested for variants. Furthermore, the lack of data broken down by country/region limits any conclusions that can be drawn regarding geographic trends in testing practices.

Strengths of our study include the large number of participants in Alnylam Act^®^, although the population in GeneAct^®^ was much smaller, and the relatively high rate of *TTR* positivity, despite the application of testing to a very broad cohort. Our findings emphasize the utility of testing in a diverse cohort and demonstrate the feasibility of confirming/excluding ATTRv through genetic testing in patients with symptoms and/or family history of ATTR. Further studies are needed to better assess the penetrance and expressivity of variants among various subgroups, for example, based on gender, ethnicity, or other potential modulating factors. Of note, the prevalence of variants in these broad cohorts with unconfirmed diagnosis and the diverse range of clinical manifestations suggests that more people than expected in the general population remain undiagnosed.

## 5. Conclusions

In this study, we have demonstrated that genetic testing is an effective approach for confirming *TTR* variants in individuals suspected of having ATTRv and for identifying asymptomatic carriers with a family history. Although barriers still exist to widespread implementation, our study highlights the benefits a robust genetic testing program can have, which ultimately has the potential to improve outcomes and quality of life in patients with ATTRv by facilitating an early diagnosis.

## Figures and Tables

**Table 1:** Baseline demographics and characteristics of participants tested in GeneAct^®^ and Alnylam Act^®^.

	Total Participants	*TTR*-Positive Participants
GeneAct^® 1^		
Participant number	*n* = 2713 ^1^	*n* = 101 ^2^
Age ^3^, years, mean (SD)	65.0 (15.9)	57.2 (18.2)
Males ^4^, *n* (%)	1730 (63.8)	57 (56.4)
Females ^4^, *n* (%)	971 (35.8)	44 (43.6)
Alnylam Act^®^		
Participant number	*n* = 89,760	*n* = 4297 ^5^
Age, years, mean (SD)	60.5 (16.5)	65.6 (14.8)
Males ^6^, *n* (%)	51,219 (57.1)	2482 (57.8)
Race ^7^, *n* (%)		
White	57,384 (63.9)	866 (20.2)
Black/African American	12,816 (14.3)	2640 (61.4)
Hispanic	3099 (3.5)	183 (4.3)
Confirmed family history, *n* (%)	2372 (2.6)	764 (17.8)

^1^ Data on family history and race are not available for GeneAct^®^. ^2^ Includes 6 participants with variants of uncertain significance. ^3^ Age was not reported for 9 participants. ^4^ Gender was not reported for 12 participants. ^5^ Participants with variants of uncertain significance (*n* = 166) are excluded. ^6^ Three participants were recorded with unknown gender. ^7^ Self-reported. SD = standard deviation.

## Data Availability

Alnylam does not share de-identified individual participant data for genetic testing programs.

## Supplementary Materials

The following supporting information can be downloaded at: https://www.mdpi.com/article/10.3390/jcm14196821/s1, Table S1: Variants identified in the TTR gene in GeneAct^®^; Table S2: Variants identified in the TTR gene in Alnylam Act^®^; Table S3: Non-TTR genes in the cardiomyopathy and neuropathy gene panels for which ≥10 participants had a positive test in Alnylam Act^®^; Table S4: Genotype–phenotype correlation in participants with selected *TTR* variants in Alnylam Act^®^; Table S5: Diagnostic yields according to individual presenting signs, symptoms, and other findings in Alnylam Act^®^; Figure S1: Distribution of variants within the TTR gene (GeneAct^®^).
