# Supplementary material for: Effectiveness and Utility of Genetic Testing in Establishing a Diagnosis of Hereditary Transthyretin Amyloidosis
Source: J Clin Med. 2025 Sep 26;14(19):6821. doi: 10.3390/jcm14196821 (PMC12524576; doi:10.3390/jcm14196821)
Supplement: Supplementary file 1 [file jcm-14-06821-s001.zip › jcm-3769433-supplementary.pdf]

Supplementary materials – Singh, A. et al.

Table S1. Variants identified in the *TTR* gene in GeneAct®.

TTR variant [1]	Participants testing positive, n	Pathogenicity
Val122Ile (p.Val142Ile)	34	Pathogenic
Val30Met (p.Val50Met)	26	Pathogenic
Arg34Ly (p.Arg54Gly)	6	Pathogenic
Ala81Thr (p.Ala101Thr)	4	Pathogenic
Cys10Arg (p.Cys30Arg)	4	Pathogenic
Ile107Val (p.Ile127Val)	3	Pathogenic
Ser77Tyr (p.Ser97Tyr)	3	Pathogenic
Val94Ala (p.Val114Ala)	3	Pathogenic
Asp74His (p.Asp94His)	2	Variant of uncertain significance
Thr60Ala (p.Thr80Ala)	2	Pathogenic
Val71Ala (p.Val91Ala)	2	Pathogenic
Ala25Ser (p.Ala45Ser)	1	Pathogenic
Arg103His (p.Arg123His)	1	Variant of uncertain significance
p.Arg5His	1	Variant of uncertain significance
Glu89Gln (p.Glu109Gln)	1	Pathogenic
Glu89Lys (p.Glu109Lys)	1	Pathogenic
(Glu7*) p.Glu27*	1	Pathogenic
Glu54Gln (p.Glu74Gln)	1	Pathogenic
Glu61Gly (p.Glu81Gly)	1	Pathogenic
Gly67Val (p.Gly87Val)	1	Pathogenic
His31Asn (p.His51Asn)	1	Variant of uncertain significance
Ser100Thr (p.Ser120Thr)	1	Variant of uncertain significance
Ser77Phe (p.Ser97Phe)	1	Pathogenic
Total	101 [2]	

[1] All in heterozygous state. [2] Includes six participants with variants of uncertain significance that were identified in symptomatic participants but that were excluded from the calculation of diagnostic yield. * indicates a stop codon.

Table S2. Variants identified in the *TTR* gene in Alnylam Act®.

TTR variant	Participants testing positive, n
**Pathogenic/likely pathogenic variants**	
Val122Ile (p.Val142Ile)	3295
Thr60Ala (p.Thr80Ala)	292
Val30Met (p.Val50Met)	271
Leu58His (p.Leu78His)	63
Phe64Leu (p.Phe84Leu)	61
Ile107Val (p.Ile127Val)	36
Ile 68Leu (p.Ile88Leu)	29
Ser77Tyr (p.Ser97Tyr)	25
Pro24Ser (p.Pro44Ser)	21
Ser50Arg (p.Ser70Arg)	18
Ala120Ser (p.Ala140Ser)	16
Asp74His (p.Asp94His)	16
Ala97Ser (p.Ala117Ser)	14
Thr60Ile (p.Thr80Ile)	13
Asp38Ala (p.Asp58Ala)	9
Ala81Thr (p.Ala101Thr)	8
Gly57Arg (p.Gly77Arg)	5
Thr40Asn (p.Thr60Asn)	5
Val20Ile (p.Val40Ile)	5
Ala109Ser (p.Ala129Ser)	4
Asp18Asn (p.Asp38Asn)	4
Ile84Ser (p.Ile104Ser)	4
Tyr78Phe (p.Tyr98Phe)	4
Val122del (p.Val142del)	4
Ala19Asp (p.Ala39Asp)	3
Arg34Gly (p.Arg54Gly)	3
Asp58His (p.Asp58His)	3
Glu89Lys (p.Glu109Lys)	3

Glu62Lys (p.Glu82Lys)	3
Glu42Asp (p.Glu62Asp)	3
His88Arg (p.His108Arg)	3
Phe33Leu (p.Phe53Leu)	3
Tyr114Cys (p.Tyr134Cys)	3
Asp38Val (p.Asp58Val)	2
Glu89Gln (p.Glu109Gln)	2
Glu54Ser (p.Glu74Ser)	2
Gly47Glu (p.Gly67Glu)	2
Thr59Lys (p.Thr79Lys)	2
Lys35Asn (p.Lys55Asn)	2
Ala36Pro (p.Ala56Pro)	1
Ala45Asp (p.Ala65Asp)	1
Ala45Thr (p.Ala65Thr)	1
Ala45Val (p.Ala65Val)	1
Ala81Val (p.Ala101Val)	1
Asp18Glu (p.Asp38Glu)	1
Asp18Gly (p.Asp38Gly)	1
Cys10Arg (p.Cys30Arg)	1
Glu89Asp (p.Glu109Asp)	1
Glu89Val (p.Glu109Val)	1
Glu54Ala (p.Glu74Ala)	1
Glu54Gln (p.Glu74Gln)	1
Gly47Val (p.Gly67Val)	1
Gly53Arg (p.Gly73Arg)	1
Gly47Arg (p.Gly87Arg)	1
Ile84Thr (p.Ile104Thr)	1
Leu32Val (p.Leu32Val)	1
Leu55Arg (p.Leu75Arg)	1
Leu55Met (p.Leu75Met)	1
Lys70Gln (p.Lys90Gln)	1
Phe44Ser (p.Phe64Ser)	1
Phe64Ser (p.Phe84Ser)	1

Ser50Gly (p.Ser70Gly)	1
Thr49Ser (p.Thr69Ser)	1
Thr59Ala (p.Thr79Ala)	1
Trp41Leu (p.Trp61Leu)	1
Val122Ala (p.Val142Ala)	1
Val20Ala (p.Val40Ala)	1
Val30Gly (p.Val50Gly)	1
Val30Leu (p.Val50Leu)	1
Val32Ala (p.Val52Ala)	1
Arg103His (p.Arg123His), Val122Ile (p.Val142Ile)	1
Ile107Val (p.Ile127Val), Val122Ile (p.Val142Ile)	1
Ile68Leu (p.Ile88Leu), p.Val122Ile (p.Val142Ile)	1
The60Ile (p.Thr80Ile), p.Val122Ile (p.Val142Ile)	1
Val30Met (p.Val50Met), Val122Ile (p.Val142Ile)	1
Total	4297 [1]
**Variants of uncertain significance**	
p.Arg5His	30
Arg103His (p.Arg123His)	17
Asp99Asn (p.Asp119Asn)	16
Ala109Thr (p.Ala129Thr)	14
Arg104Cys (p.Arg124Cys)	11
Asn27Ser (p.Asn47Ser)	6
Ala109Val (p.Ala129Val)	5
Leu82Phe (p.Leu102Phe)	5
Ala37Thr (p.Ala57Thr)	4
p.His4dup	4
Ser50Asn (p.Ser70Asn)	4
p.His4del	3

His56Arg (p.His76Arg)	3
p.Arg5Cys	2
Glu61Gly (p.Glu81Gly)	2
Gly101Ser (p.Gly121Ser)	2
His31Asn (p.His51Asn)	2
Ile68Thr (p.Ile88Thr)	2
Ala108Thr (p.Ala128Thr)	1
Ala108Val (p.Ala128Val)	1
p.Ala12Asp	1
p.Ala12Ser	1
Ala37Asp (p.Ala57Asp)	1
Arg21Gln (p.Arg41Gln)	1
Arg21Leufs*22 (p.Arg41Leufs*22)	1
Asn124Ser (p.Asn144Ser)	1
Glu42Lys (p.Glu62Lys)	1
Glu62Asp (p.Glu82Asp)	1
p.Gly13Arg	1
p.His4Leu	1
Leu82Pro (p.Leu102Pro)	1
Lys9Glu (p.Lys29Glu)	1
Pro86Ala (p.Pro106Ala)	1
Pro102Leu (p.Pro122Leu)	1
Pro125His (p.Pro145His)	1
Thr123Ala (p.Thr143Ala)	1
Thr3Met (p.Thr23Met)	1
Tyr105His (p.Tyr125His)	1
Tyr116 (p.Tyr136His)	1
Intronic	5
Gain (entire coding sequence)	4
Silent	3
Splice donor	1
Total	166

[1] 4295 with heterozygous variant and 162 with homozygous variant. * indicates a stop codon.

Table S3. Non-*TTR* genes in the cardiomyopathy and neuropathy gene panels for which ≥10 participants had a positive test in Alnylam Act®.

Cardiomyopathy		Neuropathy		Cardiomyopathy and neuropathy	
Gene	Positive, n	Gene	Positive, n	Gene	Positive, n
TTN	666	PMP22	2734	LMNA	195
MYBPC3	577	MFN2	449	GLA	53
MYH7	286	MPZ	409	BAG3	36
DMD	118	GJB1	406		
DSP	107	SPTLC1	60		
FLNC	98	SPTLC2	38		
PKP2	77	TRPV4	36		
LZTR1	65	KIF1A	34		
TNNI3	54	LITAF	25		
TNNT2	53	GDAP1	23		
ALPK3	49	SMN1	23		
SCN5A	38	SH3TC2	22		
TPM1	30	DRP2	20		
RBM20	27	HMBS	19		
SDHA	27	MORC2	19		
NF1	23	POLG2	18		
DSG2	18	ATP1A1	11		
CSRP3	15	DNM2	11		
PLN	13	RAB7A	11		
FHL1	11				
PRKAG2	10				

Table S4. Genotype–phenotype correlation in participants with selected *TTR* variants in Alnylam Act®.

Presenting signs and symptoms, n/N (%) [1]	V122I (p.V142I)	T60A (p.T80A)	V30M (p.V50M)	Other *TTR* variants
Heart disease	2443/3299 (74.1)	110/292 (37.7)	56/271 (20.7)	138/434 (31.8)
Positive imaging [2]	160/327 (48.9)	3/19 (15.8)	5/20 (25.0)	7/39 (17.9)
Sensory and motor	767/3299 (23.2)	89/292 (30.5)	142/271 (52.4)	196/434 (45.2)
Family history of ATTRv [2]	713/3299 (21.6)	194/292 (66.4)	146/271 (53.9)	258/434 (59.4)
Musculoskeletal indicators	57/327 (17.4)	2/19 (10.5)	7/20 (35.0)	7/39 (17.9)
Carpal tunnel syndrome	512/2972 (17.2)	50/273 (18.3)	33/251 (13.1)	86/395 (21.8)
Autonomic dysfunction	328/3299 (9.9)	51/292 (17.5)	38/271 (14.0)	64/434 (14.7)
Renal abnormalities	224/3222 (7.0)	4/278 (1.4)	5/258 (1.9)	7/415 (1.7)
Generalized fatigue	68/1296 (5.2)	10/147 (6.8)	5/140 (3.6)	17/218 (7.8)
Spinal stenosis/spinal radiculopathy	138/2895 (4.8)	12/259 (4.6)	17/238 (7.1)	14/376 (3.7)
Biopsy positive for amyloid [2]	148/3222 (4.6)	15/278 (5.4)	5/258 (1.9)	18/415 (4.3)
Ocular changes [3]	126/3299 (3.8)	5/292 (1.7)	16/271 (5.9)	16/434 (3.7)
Unintentional weight loss	18/1296 (1.4)	7/147 (4.8)	4/140 (2.9)	11/218 (5.0)

[1] Data taken from the test requisition forms submitted by treating healthcare professionals in Alnylam Act®. [2] Indicators of a high index of suspicion. [3] Confirmed cases of ocular amyloidosis where a pathogenic or likely pathogenic *TTR* variant is present. ATTRv = hereditary transthyretin amyloidosis.

Table S5. Diagnostic yields according to individual presenting signs, symptoms, and other findings in Alnylam Act®.

Presenting signs and symptoms [1]	Positive *TTR* result	Negative *TTR*, positive result in other gene	Positive results, overall
Family history of ATTRv [2]	27.6%	3.1%	30.7%
Biopsy positive for amyloid [2]	15.5%	0.5%	16.0%
Positive imaging [2]	14.2%	2.6%	16.8%
Unintentional weight loss	9.7%	6.1%	15.8%
Heart disease	7.7%	6.9%	14.6%
Carpal tunnel syndrome	7.0%	5.4%	12.4%
Renal abnormalities	6.1%	3.6%	9.7%
Generalized fatigue	5.4%	8.9%	14.3%
Ocular changes [3]	4.3%	5.0%	9.3%
Autonomic dysfunction	2.8%	4.1%	6.9%
Spinal stenosis/spinal radiculopathy	2.8%	3.2%	6.0%
Sensory and motor	2.0%	7.9%	9.9%
Musculoskeletal indicators	1.8%	7.3%	9.1%

[1] Data taken from the test requisition forms submitted by treating healthcare professionals in Alnylam Act®. [2] Indicators of a high index of suspicion. [3] Confirmed cases of ocular amyloidosis where a pathogenic or likely pathogenic *TTR* variant is present. ATTRv = hereditary transthyretin amyloidosis.

Figure S1. Distribution of variants within the *TTR* gene (GeneAct®).

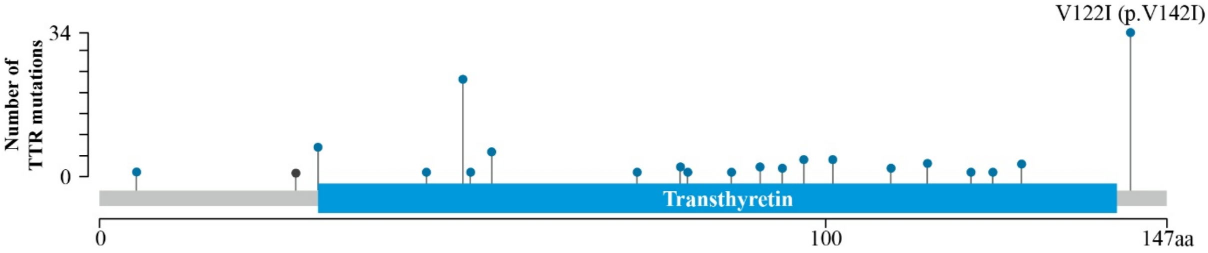

n = 101 [2]

[1] The black circle represents a stop codon in the variant. [2] Includes five variants of uncertain significance that were identified in six symptomatic participants but that were excluded from the calculation of diagnostic yield.
